# Editorial: Journey to the Center of the Brain: Cell Physiology and Intercellular Communication in White Matter

**DOI:** 10.3389/fncel.2022.864368

**Published:** 2022-03-14

**Authors:** Grace Flower, Nicola B. Hamilton, Maria Kukley

**Affiliations:** ^1^Wolfson Centre for Age-Related Diseases, Institute of Psychiatry, Psychology and Neuroscience, King's College London Guy's Campus, London, United Kingdom; ^2^Laboratory of Neuronal and Glial Physiology, Achucarro Basque Center for Neuroscience, Leioa, Spain; ^3^IKERBASQUE Basque Foundation for Science, Bilbao, Spain

**Keywords:** central nervous system (CNS), white matter, methods, diseases, omics, myelin, ion channels, neuron-glia interaction

White matter (WM) consists of myelinated axons, which propagate neuronal information over long distances to allow for connectivity between different brain areas. Previously thought to simply assist neuronal transmission, WM structure and function has recently been shown to play a larger role in normal physiology and pathology. These discoveries are the result of the emergence of new, state-of-the-art, imaging, electrophysiological, and omics technologies, allowing us to research WM in humans and animal models (Figure 1). For instance, the use of MRI has revealed changes in WM structure during learning (Sampaio-Baptista and Johansen-Berg, 2017) while single-cell electrophysiology helped discover synaptic signaling between axons and oligodendroglial cells in the WM (Kukley et al., 2007; Ziskin et al., 2007), both findings support the idea that cells within the WM respond to neuronal function. Omic approaches that analyze the gene expression profile of WM cells in great detail have revealed heterogeneity within cellular populations and suggested a link between myelination-related genes and decreased WM integrity in psychiatric disorders (Chavarria-Siles et al., 2016; Jakel et al., 2019; Safaiyan et al., 2021; Amor et al., 2022).

**Figure 1:**
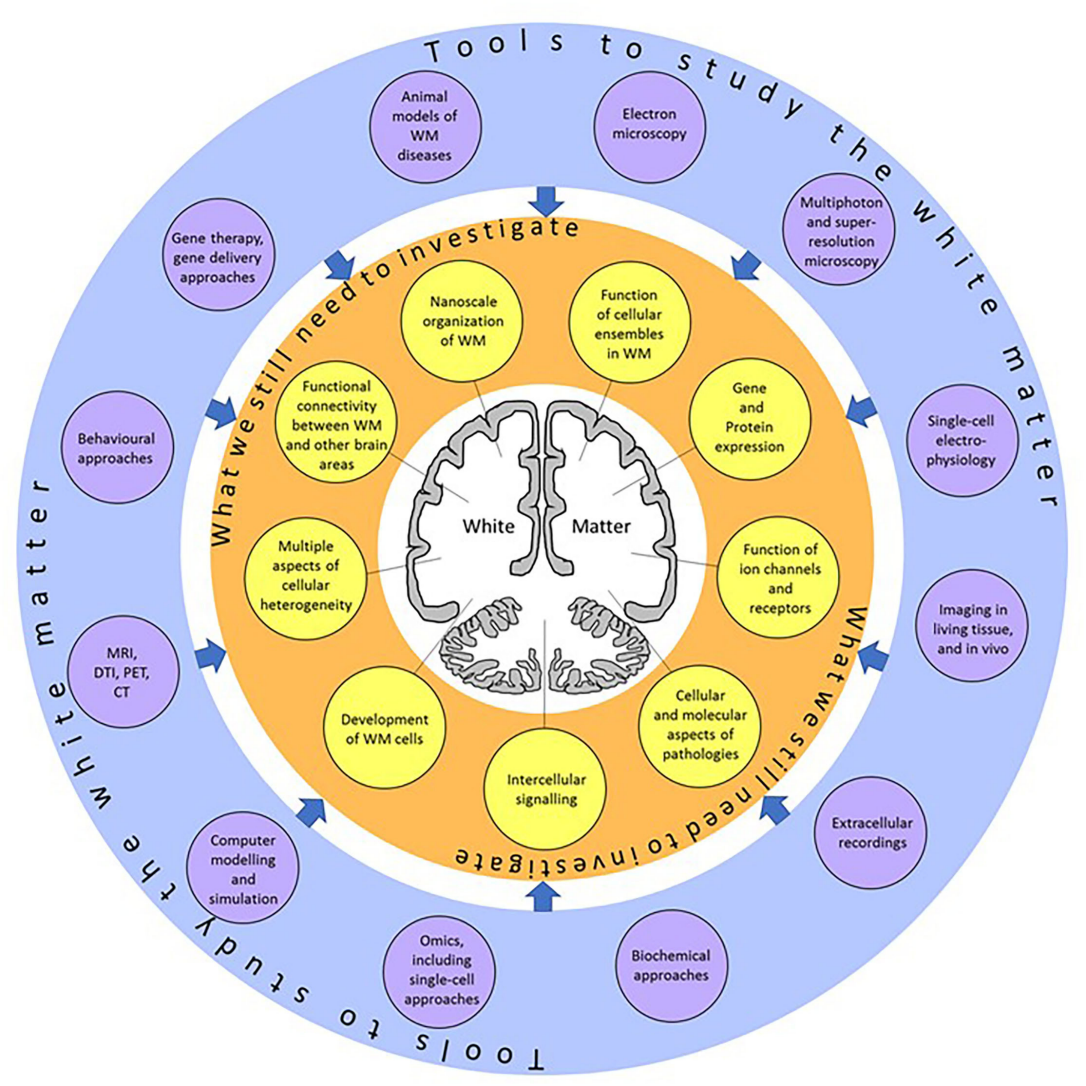
A summary of the topics (yellow circles) which still need to be investigated, and the research tools (violet circles) which can be applied to explore those topics. Using these techniques, we will better understand the physiology and function of the white matter at single-cell resolution.

The Research Topic “Journey to the Center of the Brain: Cell Physiology and Intercellular Communication In White Matter” encompasses literature reviews and research articles describing recent achievements in WM research at the single-cell level, emphasizing the need for further studies of physiology and the structure of WM cells.

Through an in-depth review, Jakel and Williams present current data from single-cell technologies and indicate which of these approaches will best answer the remaining questions in human WM pathologies. This review hints at an exciting future where we will gain a deeper understanding of disease and create diagnostic and therapeutic tools. To utilize the powerful applications of single-cell technologies, Jahn et al. revisited the myelin proteome of mice, using mass spectrometry to quantitatively measure the most abundant myelin proteins (PLP, MBP, CNP, and MOG) and determine the complexity of the less abundant myelin proteins. Interestingly, they identified that the myelin proteome displays minor changes if assessed 6 h post-mortem. From this, they formed a resourceful dataset for anyone studying CNS myelin, be that in mouse models of demyelination or clinical WM disorders. Taking the myelin and axonal proteome to a structural and mechanical level Shimizu et al. used a unique tension sensor based on fluorescence resonance energy transfer to investigate the physical factors involved in myelin formation in response to axon diameter. The authors observed higher tension at oligodendrocyte processes that contact larger diameter axons and longer oligodendroglial focal adhesions on these axons. These data suggest that oligodendrocytes physically respond to axon diameter by changing their cytoskeletal organization and myelin formation. This is an extremely valuable finding in understanding WM physiology.

Through a comprehensive review of published bulk and single-nuclei RNA-sequencing datasets, Boscia et al. presented the expression levels of ion channels in multiple sclerosis (MS). The authors show there is an upregulation of voltage-gated Na^+^ channel genes and downregulation of voltage-gated Cl^−^ channels in WM lesions. This supports the imbalance of Na^+^ homeostasis and the increased excitability observed in progressive MS. On the other hand, there is an upregulation of voltage-gated K^+^ channel genes that could be a protective function in response to the altered Na^+^/Cl^−^ homeostasis. To complement these findings, Serrano-Regal et al. reviewed the role of GABAergic signaling in myelinating cells and its potential for developing therapies in demyelinating disease. They detailed the expression levels of GABA receptors in the myelinating cells with support of functional data demonstrating the involvement of GABAergic signaling in precursor cell development and maturation of myelinating cells. This review supports further study into targeting GABAergic signaling in WM pathology.

Maas and Angulo reviewed the potential therapeutic approach of enhancing neuronal activity to improve remyelination in MS. The authors discussed pre-clinical and clinical evidence that supports the notion that neuronal stimulation induces myelination and behavioral changes. Clinically, non-invasive brain stimulation has been shown to reduce or ameliorate MS symptoms. Maas and Angulo support this attractive therapeutic approach by further discussing the neurobiological basis of enhancing neuronal activity at the single-cell level.

From functional to structural, Dutta and Fields present interesting findings on the myelin membrane protein complex, NF155, which attaches the myelin to the axon in the paranodal region. Using Crispr-Cas9 gene editing, they identified a thrombin binding site of the NF155 complex that is implicated in the plasticity of the myelin sheath. When this site is deleted, there is disruption to myelination, nodal and paranodal organization. Mice with this deletion present tremor and ataxia, demonstrating the importance of this domain for the stability of the protein complex and therefore myelin-axonal integrity. On the note of myelin plasticity, Liu et al. reviewed and developed a model for myelin plasticity in the neural circuits underlying threat and safety learning. These stimuli are a major cause for the emergence of anxiety-related disorders and understanding the circuitry dynamics underlying the response to these stimuli will better inform us on the development of these psychiatric disorders. This review suggests further study is needed to understand myelin plasticity in psychiatric disorders.

Lastly, Cocola et al. share interesting findings on vasculature function in glioblastoma. After recently identifying the transmembrane protein, TMEM230, as a regulator of development associated with angiogenesis in the zebrafish (Carra et al., 2018), the authors further explored its role in the pathogenesis of glioblastomas. They found TMEM230 was necessary for cell growth in a cellular model of human glioblastoma and observed high TMEM230 levels in low-grade gliomas in the patients. These findings suggest that downregulation of TMEM230 expression may inhibit low-grade glioma and glioblastoma tumor progression while supporting the normal formation of blood vessels. As TMEM mRNA is expressed in the WM (according to The Human Protein Atlas) and TMEM230 is necessary for the formation of WM during early development. Future research on the role of TMEM230 in the WM will be of specific interest.

In conclusion, the future looks bright for research into understanding WM structure and function. There is a clear need for further studies of the WM at single-cell resolution in both animal models and human patients but the technologies are becoming more accessible and fast-evolving to enable this research to take place (Figure 1). It is becoming more obvious that the WM is more than just passive tissue. The functional properties of the cells forming the WM can influence brain function and may provide therapeutic targets for WM pathologies.

## Author Contributions

GF, NH, and MK contributed to this editorial by summarizing the findings and content of all scientific manuscripts included in the Research Topic. GF prepared the original draft of the manuscript, MK prepared the figure. All authors approved the submitted version of the manuscript.

## Funding

Funding of NH and GF was from the European Leukodystrophies Association International (ELA2017-015I4), the Medical Research Council (MR/S003045/1 and MR/V000470/1), and King's College London. GF is supported by the UK Medical Research Council MR/N013700/1 and King's College London member of the MRC Doctoral Training Partnership in Biomedical Sciences. The work of MK was supported by the Ikerbasque (Basque Foundation for Science), the Spanish Ministry of Science and Innovation (grant PID2019-110195RB-I00), and the Basque Government PIBA Project (PIBA 2020_1_0030).

## Conflict of Interest

The authors declare that the research was conducted in the absence of any commercial or financial relationships that could be construed as a potential conflict of interest.

## Publisher's Note

All claims expressed in this article are solely those of the authors and do not necessarily represent those of their affiliated organizations, or those of the publisher, the editors and the reviewers. Any product that may be evaluated in this article, or claim that may be made by its manufacturer, is not guaranteed or endorsed by the publisher.
